# Microbial Quality, Risk Analysis, and Molecular Detection of *Escherichia coli* O157 and *Salmonella* spp. in Fresh Raw Beef Sold in Ho Central Market, Ghana

**DOI:** 10.1155/ijfo/8395815

**Published:** 2026-06-26

**Authors:** Felix Kwashie Madilo, Emmanuel Letsyo, Nii Korley Kortei, Amy Atter, Favour Odukoya, Liticia Effah-Manu, Emmanuel Bassau Quansah, Christopher Galley

**Affiliations:** ^1^ Department of Food Science and Technology, Ho Technical University, Ho, Volta Region, Ghana, htu.edu.gh; ^2^ Department of Nutrition and Dietetics, School of Allied Health Sciences, University of Health and Allied Sciences, Ho, Ghana, uhas.edu.gh; ^3^ Food Microbiology and Mushroom Division, CSIR-Food Research Institute, Accra, Ghana, foodresearchgh.org

**Keywords:** contamination, food pathogens, fresh beef, poor hygiene, *Salmonella*

## Abstract

Beef is a prominent dietary component in many developing countries, including Ghana, due to its rich protein, energy, and fat contents. However, it has become a significant source of foodborne pathogens, including *Escherichia coli* O157 and *Salmonella*, which can lead to severe gastrointestinal infections. This study investigated food hygiene practices and the microbial safety of beef sold at the Ho Central Market in Ghana. Sixty‐three beef handlers and processing facilities in 25 shops were conveniently sampled using a structured questionnaire. Twelve beef samples were also collected in duplicates at 9:00 a.m. and 3:00 p.m. from the market and subjected to microbial analyses using standard procedures (aerobic plate count [NMKL No. 86, 2013], coagulase‐positive staphylococci [NMKL No. 66, 5th Ed., 2009], Enterobacteriaceae [NMKL 144, 2005], *E. coli* O157 [NMKL 125, 2005], and *Salmonella* [NMKL No. 71, 1999]). The data collected were analyzed using SPSS Version 22. The food hygiene protocol observation study revealed that food safety and hygiene standards were compromised. However, personal hygiene practices like wearing suitable PPE (100%), using standard protocols for handwashing (100%), and removing pieces of jewelry before processing (92%) were observed. For microbial analyses, the results showed that almost all the beef samples were highly contaminated under each indicator, except the sample from shade Vendor 1 at 9:00 a.m., which recorded Enterobacteriaceae (3.18 ± 5.00) and *E. coli* (2.68 ± 4.04) below Ghana′s Food and Drug Authority thresholds. While almost all the samples (75%) were free from *E. coli* O157, the *Salmonella* species contaminated about 75% of the samples. However, there are no statistical differences between the samples collected at 9:00 a.m. and those at 3:00 p.m. (*p* > 0.05). The presence of high microbial counts, *E. coli* O157, and *Salmonella* in beef samples could be attributed to inadequate and substandard infrastructure. Hence, effective food safety measures and regular monitoring are imperative to ensure the safety of beef products and protect public health.

## 1. Introduction

Beef has many nutritional contents, including protein, vitamins, fats, and minerals, and therefore plays a significant role in human diets [1, 2]. The demand for beef in developing countries like Ghana has increased over the years as the production and consumption pattern of meat has increased significantly with the availability of income [1, 3]. However, beef could be a major channel for the transfer of foodborne pathogens to humans. The contamination of beef by microbial pathogens might occur at different stages in the processing chain [1]. The different stages of contamination include preslaughter, slaughter, and postslaughter handling [4]. The contamination can occur when there are regular body and facility contacts and exposure to water and air during processing [5]. Hence, adopting stringent safety and hygienic protocols during processing and production is required. The US Centers for Disease Control and Prevention (USCDC) documented about 839 foodborne disease outbreaks resulting in 14,972 illnesses, 794 hospitalizations, and 17 deaths [6]. A study in Egypt on bacterial contamination of beef carcass preparation at an abattoir exceeded the standard limit of 10^6^ CFU/cm^2^ [7]. In India, a similar study on the microbiological quality of meat sold in slaughterhouses and retail shops had a varying prevalence for *Salmonella* spp. and *Escherichia coli* [8]. Herrera‐León et al. [5] in Mexico detected about 11% of *Salmonella* spp. in the beef samples analyzed. Similarly, in a study by Gomes et al. [9] in Brazil, 7.4% of the beef samples tested positive for *Salmonella* spp. In most developing countries like Ghana, the majority of the slaughterhouses are substandard and lack modern infrastructure; poorly designed substandard equipment is used. This may result in the contamination of beef and beef products, leading to food poisoning and other health‐related problems. In a study on the bacteriological and parasitic quality of beef sold in the Ashaiman Market, it was reported that the beef samples were contaminated with *Salmonella* Typhimurium, *E. coli*, and *Staphylococcus aureus* [10]. Adjei et al. [10] concluded that the contamination of the raw beef was a result of unhygienic practices and poor sanitary conditions at the abattoirs and retail shops. Ofori‐Boadu et al. [11] analyzed 125 beef samples for *Salmonella* contamination and reported a prevalence rate of about 15%. Tano‐Debrah et al. [12] investigated the prevalence and antibiotic resistance profiles of *Salmonella* isolates from beef and pork samples obtained from Kumasi and documented that about 19% were positive for *Salmonella* contamination. Also, in the Northern, Central, and Greater Accra Regions of Ghana, different prevalent rates of *E. coli* and other pathogens in beef have been documented and reported [1, 10, 13–15]. Despite these numerous studies on microbial pathogen contamination of meat, there is limited information, particularly on *Salmonella* and *E. coli* contamination of beef in the main markets of Ghana. To the best of our knowledge, the prevalence of pathogenic *E. coli* (*E. coli* O157:H7) in beef is yet to be reported in Ghana, even though most of the global foodborne disease outbreaks were attributed to *E. coli* O157:H7 contamination [16]. Hence, this investigation was designed to evaluate and characterize the pathogenic *E. coli* O157 and *Salmonella* species in the two main raw and fresh beef markets of Ghana.

## 2. Materials and Methods

### 2.1. Study Area Description

Ho Municipal Assembly is the administrative and commercial center of the Volta Region of Ghana. It shares boundaries with the Ho West District to the North and West, the Adaklu District to the South, and Togo to the East. The area has a tropical climate and a gently hilly landscape shaped by the Akwapim–Togo ranges. The municipality has about 190,000 people according to the 2021 Population and Housing Census [17]. Ho municipality is a major hub for education, health, and commerce and hosts institutions such as Ho Technical University (HTU) and the University of Health and Allied Sciences (UHAS). The economy of the municipality is driven by trading, public administration, agriculture, and small‐scale industries. Culturally, it is predominantly inhabited by the Ewes and is known for events such as the Asogli Yam Festival.

### 2.2. Study Site and Sampling Techniques

Abattoir chains in Ho are an organized process involved in slaughtering animals, inspecting carcasses, dressing meat, and distributing it to markets and consumers. The chains operate basically through municipal slaughter slabs. It is important to prevent contaminated or diseased meat from entering the food supply chain, maintain high meat hygiene and quality standards during slaughter and handling, and ensure safe meat is supplied to the markets, restaurants, and households. On average, there were about seven vendors per butcher shop (shade). A two‐stage cluster sampling technique was used to collect beef samples from Ho municipality for microbial analysis. The clusters were the beef distribution centers, such as abattoirs and butcher shops. The locations represent where consumers obtain beef and where contamination risks may vary due to differences in hygiene and handling practices. In this method, the total population of butchers (115) was divided into clusters based on similar characteristics they possessed. The clusters for the study and the participants from the selected clusters were randomly assigned. Clustering allowed the authors to first select major distribution centers and then randomly sample beef within them, ensuring representativeness while reducing cost and logistical constraints. However, a convenient sampling technique was adopted for the observation studies. The processing facilities observed were scored “1” for presence, “0” for absence, and “poor” for presence but defective. Final scores less than 50% represent deficiencies that might pose significant public health risks (high risks), while scores of 50% and above demonstrate the presence of essential control measures necessary for safe food production (acceptable to low risks).

### 2.3. Sample Size and Data Collection

A structured questionnaire (observation checklist adopted from Fasanmi et al. [3] with modification) was developed and thereafter validated by food safety experts from HTU, UHAS, Ho, Volta Region, and the Food Research Institute (FRI), Accra. The questionnaire, which has three sections, such as Section “A” (demographic characteristics), Section “B” (hygiene status of facilities and equipment), and Section “C” (food safety and hygiene indicators), was piloted among five meat vendors in Ho township. The difficulties raised by the retailers were used to improve the observation checklist before rolling it out for data collection. The questionnaire was used to sample 63 beef vendors and some processing facilities (fridges and freezers, cutting instruments, washing and drainage facilities, working platforms, etc.) in 25 processing shops for data collection using a convenient technique. A total of 12 beef samples were collected in duplicates from Ho Central Market at 6‐h intervals (9:00 a.m. and 3:00 p.m.) using a random sampling technique, placed in sterile stomacher bags, and transported on ice to the FRI, Microbiology Laboratory, Accra, for analysis.

### 2.4. Eligibility Criteria

The respondents must answer “yes” to all screening questions before progressing to Q1:

a. I confirm that I have understood the purpose and rationale for the study and have asked and received answers to any questions raised.
b. I understand that my participation is voluntary and that I am free to withdraw at any time without giving a reason and without my rights being affected in any way.
c. I understand that the researchers will hold all information and data collected securely and in confidence and that all efforts will be made to ensure that I cannot be identified as a participant in the study. I, therefore, agree to take part in the above study.
d. I confirm that I am a meat processor/retailer in Ho municipality.
e. I have read and understood all the above statements regarding my participation in this study, and I am willing to give my consent to participate in this study. I have not waived any of my rights by signing this consent form. Upon signing this consent form, I will receive a copy for my personal records.

### 2.5. Microbial Enumeration and Characterization

All the microbial enumeration and characterization were done using standard techniques. Aerobic plate count (APC) was conducted according to procedures described by NMKL No. 86, 2013, *Staphylococcus aureus* (NMKL No. 66, 5th Ed., 2009), Enterobacteriaceae (NMKL 144, 2005), *E. coli* O157 (ISO 16654:2001), and *Salmonella* (NMKL No. 71, 1999). Briefly, about 10 g of the beef sample was weighed using the electronic balance (Model V22PWE3T) into the stomacher bag. About 90 mL of salt peptone solution (SPS) (Merck) was added to the sample. The stomacher bag was then placed in the stomacher machine (Model 400 Circulator, London, England) and homogenized for 30 s. Using SPS, a sixfold serial dilution was prepared, and a pour plate technique was adopted to enumerate APC on Plate Count Agar (PCA) (Merck) and Enterobacteriaceae on Violet Red Bile Glucose Agar (VRBGA) (JO 2049S, CDH), and the setups were incubated at 37°C for 24 h. After incubation, the suspected visible colonies were counted using the colony counter (Nr 1000600UK/3091). Suspected colonies for Enterobacteriaceae were purified and confirmed by the oxidase strip test.

For coagulase‐positive staphylococci, determination was done according to procedures described by NMKL No. 66, 5th Ed. (2009). About 10 g of the beef sample was weighed into the stomacher bag. About 90 mL of SPS was added and homogenized for about 30 s. Baird–Parker (BP) Agar with egg yolk tellurite emulsion (Oxoid SR0054C) and Blood Agar were prepared and allowed to solidify. Appropriate serial dilutions were spread‐plated and then incubated in an inverted position at 37°C for 44 ± 3 h. Suspected coagulase‐positive staphylococcal colonies were identified by the presence of black or gray, shining, and convex colonies, which were surrounded by a clear halo zone due to lecithinase activity. These colonies were then purified onto Nutrient Agar (NA) and incubated at 37°C for 24 h. Gram test, hemolytic test, catalase test, and coagulase tests using rapid rabbit fibrinogen were performed to confirm the presence of coagulase‐positive staphylococcal species in the beef samples, in accordance with the procedures outlined in NMKL No. 66, 5th Ed. (2009).

For *E. coli*, the samples were pour‐plated using Tryptone Soya Agar (TSA) (TM 017, SRL). After the agar was allowed to solidify, the Violet Red Bile Agar (VRBA) was overlaid. After setting, the plate was incubated at 44°C (NE9‐112S, England) for 48 h. The suspected visible colonies were then counted. Thereafter, the individual colonies were placed in *E. coli* broth (M1426, HiMedia) and incubated for 24 h. Gas formation in the Durham tubes indicated the presence of coliforms in the sample. About 1 mL of the sample was then introduced into 9 mL of tryptone broth (TM 017, SRL) and incubated at 44°C for 24 h, and an indole test was conducted by adding Kovac′s reagent. The formation of the brown ring on the surface of the solution indicated the presence of *E. coli* O157 in the samples.

For *Salmonella* detection, about 25 g of the beef sample was inoculated into about 225 mL of Buffered Peptone Water (BPW) (Oxoid). The solution was then incubated at 37°C (NE8‐240S, England) for 24 h. One milliliter of the sample was then introduced into 9 mL of RV (Rappaport–Vassiliadis) in tubes, vortexed, and incubated at 42°C using a water bath (Nickel‐Electro, Weston‐super‐Mare, Somerset; 111202) for 24 h. After the incubation, a loop of the sample was taken and streaked on XLD (xylose lysine deoxycholate) (CM0469, Oxoid) plates and incubated at 37°C for 24 h. The suspected *Salmonella* colonies that were black, shiny, and rod‐shaped were identified and streaked on NA and incubated for 24 h. Colonies with a black center were purified on nonselective media and incubated at 37°C for 24 h for further confirmation tests.

### 2.6. Confirmation Studies

The suspected colonies (six each) of *E. coli* O157 and *Salmonella* spp. isolated from the samples were randomly selected and cultured in Brain Heart Infusion broth for 24 h. About 1 mL of suspension of each of the broth cultures was transferred into 2‐mL microcentrifuge tubes. The DNA was purified from the cultures using a GenElute genomic DNA extraction kit (Sigma‐Aldrich, Mumbai, India) as stated in [18]. The rfbE gene was targeted to distinguish *E. coli* O157 from other *E. coli* serotypes because it is involved in the biosynthesis of the O157‐specific lipopolysaccharide (LPS) O‐antigen, a major surface antigen used in serological classification. Specifically, the rfbE gene encodes perosamine synthetase, an enzyme required for the synthesis of perosamine, a sugar component unique to the O157 O‐antigen. Since the rfbE gene is highly conserved among O157 strains and absent in non‐O157 *E. coli*, its detection by PCR serves as a reliable molecular marker for the confirmation of *E. coli* O157 isolates. Consequently, amplification of the rfbE gene enhances the specificity of pathogen identification and helps differentiate O157 strains from other pathogenic and nonpathogenic *E. coli* variants [19]. Amplification of the ompC gene coding for an outer membrane protein for the detection of *Salmonella* species has a higher level of specificity than conventional methods [20]. Amplification reactions for the detection of *Salmonella* spp. were carried out in a 25 *μ*L volume comprising 12.5 *μ*L Quick‐Load 2X Master Mix, 1 *μ*L each of 10 *μ*mol forward and reverse primers (Table 1), 3 *μ*L of template, and 7.5 *μ*L of Ampliqon PCR grade water for each sample. The cycling conditions comprised an initial denaturation of 95°C for 5 min, a 38‐cycle step of 94°C for 30 s, 55°C for 1 min, and 72°C for 3 min, followed by a final extension step of 72°C for 10 min. PCR products were resolved in a 1.5% agarose gel stained with ethidium bromide at 80 V for at least 1 h. The resolved products were illuminated and photographed via a Quantum Vilber Lourmat gel documentation system.

**Table 1:** The primers used for molecular characterization of *E. coli* and *Salmonella*.

Organism	Primer	Sequence (5′‐3′)	Reference
*E. coli*	Ec1‐F	TGT CCA TTT ATA CGG ACA TCC ATG	[20]
	Ec2‐R	CCT ATA ACG TCA TGC CAA TAT TGC C	
*E. coli* O157:H7	Ec3‐F	GCG CTG TCG AGT TCT ATC GAG C	[20]
	Ec4‐R	CAA CGG TGA CTT TAT CGC CAT TCC	
*Salmonella* spp.	Sm1‐F	ATC GCT GAC TTA TGC AAT CG	[21]
	Sm2‐R	CGG GTT GCG TTA TAG GTC TG	

### 2.7. Statistical Analysis

Statistical analyses were performed using the Statistical Package for the Social Sciences (SPSS) Version 22. Descriptive statistics were used to determine the means and standard deviations for the variables measured. One‐way analysis of variance (ANOVA) was used to determine the differences that existed among groups, and the statistical significance was accepted at *p* < 0.05. Results are presented as mean ± standard deviation. Graphs were prepared using Microsoft Excel.

## 3. Results and Discussion

### 3.1. Demographic Characteristics (Oral Interview)

**Figure 1:**
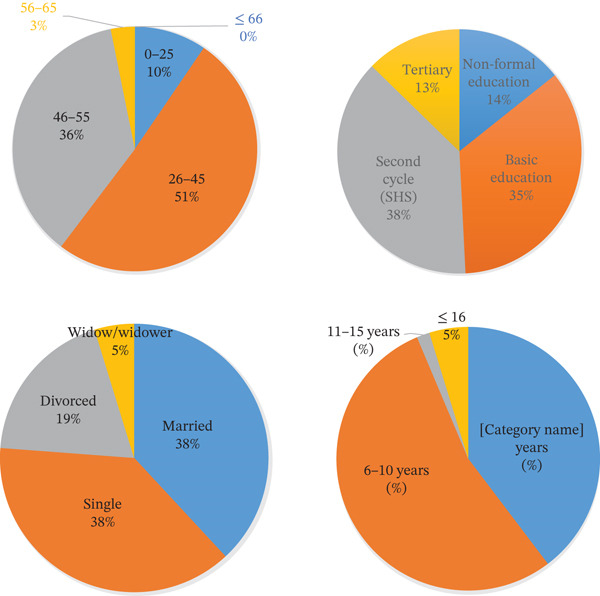
The demographic characteristics of the meat vendors in Ashaiman and Ho central markets. (A) Year (age). (B) Educational level. (C) Marital status. (D) Duration in business.

Demographically, the results indicated that all the respondents (100%) were male. The majority were between the ages of 26 and 45 years (51%), held a second cycle education certificate (38%), were either single or married (38%), and had been in the meat processing and vending business for 6–10 years (54%) (Figure 1). This is very important due to the fact that the higher the educational level of the vendors and the longer they stay in the food processing business, the more experience they have that might contribute to better food safety and hygiene knowledge of the vendors [22]. Again, as many as about 54 (86%) did not receive any form of training or workshops on the principles of food hygiene and safety. The remaining nine (14%) stated that they attended only good hygiene practice (GHP) training when they were asked to do so. Food safety, hygiene, and sanitation training are mandatory for all food handlers, including commercial and household food handlers [23]. This would enable them to avoid microbial cross‐contamination and produce food that is safe and healthy at all times across the food production chain [24].

### 3.2. Observation of Facilities and Other Equipment in the Study Areas

After the data on safety and hygienic conditions of facilities and other equipment used in the 25 shops were collected and analyzed, the results (Table 2) reveal that apart from having good standard walls (88%) and fair standard doors and doorways (56%), the hygiene conditions of the majority of the facilities and equipment were very poor. The safety and hygiene standards of most facilities and equipment in most vending shops were severely compromised. Specifically, some of these facilities and equipment include working platforms, cold chain systems, drainage facilities, sinks, basins, and floors (Table 2).

**Table 2:** Hygiene facilities and equipment observed (25 shops) (Fasanmi et al. [3]).

Facilities and equipment observed	Score < 50	Score ≥ 50	Remark
Working platform	20 (80)	5 (20)	Poor
Implement sterilizer	25 (100)	0 (0)	Poor
Refrigerators and freezers	13 (52)	12 (48)	Poor
Drainage facilities	16 (64)	9 (36)	Poor
Handwashing facilities (basins)	15 (60)	10 (40)	Poor
Presence of detergents for washing hands	14 (56)	11 (44)	Poor
Towels/tissues for drying hand	20 (80)	5 (20)	Poor
Adequate toilet facilities available	25 (100)	0 (0)	Poor
Sink with running water (cold and hot)	25 (100)	0 (0)	Poor
Use a screen to protect the meat from flies	10 (40)	15 (60)	Poor
Standard floors (tiles, smooth concrete, or waterproof material)	16 (64)	9 (36)	Poor
Standard wall (glazed tile, smooth cement plaster)	3 (12)	22 (88)	Good
Standard doors and doorways	11 (44)	14 (56)	Fair
Standard water supply system	13 (52)	12 (48)	Poor
Lighting system available (candlelight)	16 (64)	9 (36)	Poor

*Note:* Scores: nonexistent to poor (0%–49%) or < 50; good to very good (50%–100%) or ≥ 50.

The use of compromised hygiene and sanitation protocols, facilities, and equipment in food processing and production centers is a recipe for microbial foodborne disease outbreaks, as a result of microbial cross‐contact and contamination. Studies by Amoah et al. [25] and Boateng and Yeboah [26] indicated that food vendors in Accra, Tamale, and Kumasi have inadequate and poor handwashing facilities. In other studies, cutting boards, knives, and food‐contact surfaces were found to be contaminated with several microbial pathogens [27, 28]. Jansen et al. [29, 30] and Rinn et al. [2] stated that improper sanitary conditions of meat preparation facilities can easily lead to pathogenic microbial contamination, including *Salmonella* spp., Shiga toxin–producing *E. coli*, and *Listeria* monocytogenes.

### 3.3. Hygiene and Sanitation Practices Observed

Safety, hygiene, and sanitation indicators were used as observation tools to analyze the personal hygiene and sanitation status of meat handlers in the two study areas. The results show that several hygiene and sanitation standards were compromised (Table 3). The water used was dirty and was at ambient temperature throughout the centers. The beef was transported from the slaughterhouse by unhygienic vehicles without a cover. Moreover, there were poor or no toilet facilities during the observation studies across the study centers. However, it was heartwarming and very commendable to observe all the handlers maintaining excellent personal cleanliness (100%), wearing suitable PPE (100%), using standard protocols for handwashing (100%), and removing pieces of jewelry (92%) before processing and selling meat (Table 3). Even though this finding is commendable, it is not enough to enhance safe and hygienic food production until other safety indicators are properly maintained.

**Table 3:** The food safety and hygiene indicators observed in the study areas (Fasanmi et al. [3]).

Safety and hygiene indicators	Score < 50	Score ≥ 50	Remark
Meat handlers maintain adequate personal cleanliness	0 (0)	63 (100.0)	Good
Handlers wear suitable personal protective equipment (PPE) to protect against contamination	0 (0)	63 (100.0)	Good
PPE is kept away from meat preparation areas	42 (66.7)	21 (33.3)	Poor
Vendors remove pieces of jewelry that might contaminate meat	12 (8.0)	43 (92.0)	Good
Employees wash hands thoroughly before and after handling meat	0 (0)	63 (100.0)	Good
Gloves are made from impermeable material and maintained in clean, sanitary conditions	62 (98.4)	1 (1.6)	Poor
Vendors confined eating, drinking, gum chewing, and use of tobacco to areas where meat is not exposed	0 (0)	63 (100.0)	Good
Waste containers are operated and handled in a manner that prevents contamination	10 (15.9)	53 (84.1)	Good
Standard procedures for cleaning and sanitizing equipment are adopted	40 (63.5)	23 (36.5)	Poor
Cleaning and sanitizing agents and other hazardous chemicals are kept in their original containers and stored appropriately	54 (85.7)	9 (14.3)	Poor
Meat‐contact surfaces used are dried and in a sanitary condition at the time of use	36 (57.1)	27 (42.9)	Poor
Single‐service articles (paper cups, towels) are stored, used, and disposed of appropriately	62 (98.4)	1 (1.6)	Poor
The water supply is from approved sources and of quality for its intended uses	63 (100.0)	0 (0)	Poor
The water temperature and pressure are maintained at suitable levels for the intended use	63 (100.0)	0 (0)	Poor
Meat processing conditions and parameters are properly controlled and monitored	60 (95.2)	3 (4.8)	Poor
Work in progress is handled and protected against microbial contamination and growth	60 (95.2)	3 (4.8)	Poor
Meat is protected against microbial contamination during transportation from slaughterhouses	63 (100.0)	0 (0)	Poor
Equipment and containers are handled and maintained to avoid cross‐contamination	40 (63.5)	23 (36.5)	Poor
Vehicles are properly designed, maintained, and kept in sanitary conditions	63 (100)	0 (0)	Poor
Equipment and containers used to process meat are constructed, handled, and maintained during processing to avoid cross‐contamination	59 (93.7)	4 (6.3)	Poor
Working floors are regularly washed and sanitized before or after work	62 (98.4)	1 (1.6)	Poor
Scratching the head and other parts of the body during meat processing and selling	62 (98.4)	1 (1.6)	Poor
Meat processing areas are effectively separated from other operations	49 (77.8)	14 (22.2)	Poor
Floors, walls, and ceilings are constructed to facilitate adequate cleaning and repair	47 (74.6)	16 (25.4)	Poor
Are there adequate lights in all handwashing, toilet areas, dressing, and locker rooms?	63 (100.0)	0 (0)	Poor
Air quality and ventilation are adequate to prevent contamination	56 (88.9)	7 (11.1)	Poor
The facility openings are protected by adequate screening to eliminate insects and rodents	53 (84.1)	10 (15.9)	Poor
Plumbing is adequately designed, installed, and maintained to prevent contamination	63 (100.0)	0 (0)	Poor
Paths, yards, and parking lots are maintained to prevent sources of contamination	63 (100.0)	0 (0)	Poor
Adequate toilet facilities are provided, equipped, and maintained clean and in good repair	63 (100.0)	0 (0)	Poor

*Note:* Scores: nonexistent to poor (0%–49%) or < 50; good to very good (50%–100%) or ≥ 50.

In light of this result, Rani et al. [31] and Ncoko et al. [32] observed that even though the beef handlers maintained standard personal hygiene, sanitizers were not used for decontamination after touching meat, and this act could contribute to microbial contamination. The high loads of bacterial pathogens were a result of a lack of good manufacturing and handling practices and sanitary standard operating protocols along the production chain [33]. Al‐Amin et al. [34] opined that unhygienic practices during meat processing could lead to different forms of microbial contamination, including *E. coli* and *Salmonella*. Olu‐Taiwo et al. [1] and Rani et al. [31] also alleged that beef and beef products could be contaminated along the processing chain by personnel and equipment during transportation.

### 3.4. Microbial Enumeration and Identification

**Figure 2:**
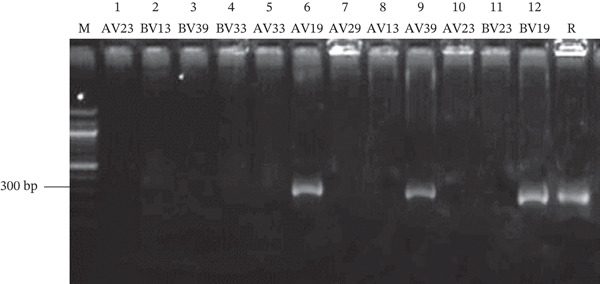
Amplification of the rfbE gene (?296 bp) for the detection of *Escherichia coli* O157. Key: A = Shade A; B = Shade B; V1, 2, or 3 = Vendors 1, 2, or 3; 9 or 3 = sample collected at 9:00 a.m. or 3:00 p.m.

**Figure 3:**
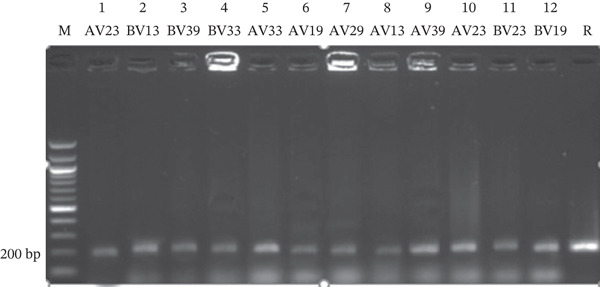
Amplification of the ompC gene (204 bp) showing slight polymorphism for the detection of *Salmonella* spp. Key: A = Shade A; B = Shade B; V1, 2, or 3 = Vendors 1, 2, or 3; 9 or 3 = sample collected at 9:00 a.m. or 3:00 p.m.

The study also carried out some analyses on several microbial indicators such as APCs, Enterobacteriaceae, *E. coli*, and coagulase‐positive staphylococci. The results presented in Table 4 reveal that almost all beef samples collected from Ho Market were highly contaminated under each indicator, except Sample BV19, which recorded Enterobacteriaceae (3.18 ± 2.00 log_10_ CFU/g) and *E. coli* (2.68 ± 1.04 log_10_ CFU/g) below the Ghana Standards Authority (GSA) thresholds [35]. The most contaminated samples were AV33 (APC [7.79 ± 4.04 log_10_ CFU/g]), BV39 (staphylococci [4.71 ± 3.51 log_10_ CFU/g]), AV19 (Enterobacteriaceae [5.92 ± 3.51 log_10_ CFU/g]), and AV39 (*E. coli* [5.72 ± 3.51 log_10_ CFU/g]). Again, the results indicated that there were no statistical differences between the samples collected at 9:00 a.m. and those at 3:00 p.m. (*p* > 0.05). From the results of molecular characterization (Figure 2), apart from AV19, AV39, and BV19 samples, which recorded the presence of *E. coli* O157, the rest of the samples were free from *E. coli* O157 contamination. Interestingly, all these contaminated samples were collected in the morning (9:00 a.m.) when the temperature of the beef was assumed to be low. For *Salmonella* species contamination, only three samples (BV39, BV13, and BV33) from Shade “B” did not record the presence of *Salmonella* species (Figure 3). However, all the samples from Shade “A” were heavily contaminated with *Salmonella* species (Figure 3).

**Table 4:** Bacterial count and standard deviation of beef from different sheds.

	Bacterial counts (log_10_ CFU/g)					
Sample	APC	*S. aureus*	Enteric	*E. coli*	*E. coli* O157	*Salmonella*
AV19	7.62 ± 3.06^a^	4.63 ± 4.00^b^	5.92 ± 3.51^d^	5.69 ± 4.03^c^	Detected	Detected
AV29	7.59 ± 6.03^b^	4.46 ± 3.51^a^	5.82 ± 4.73^ab^	5.23 ± 3.00^b^		Detected
AV39	6.95 ± 4.00^c^	4.44 ± 3.51^b^	5.78 ± 2.52^ab^	5.72 ± 3.51^a^	Detected	Detected
AV13	7.69 ± 5.13^c^	3.84 ± 3.06^a^	5.73 ± 4.13^b^	5.48 ± 4.51^ab^		Detected
AV23	7.71 ± 4.51^b^	4.44 ± 2.51^b^	5.38 ± 4.00^ab^	5.34 ± 3.61^a^		Detected
AV33	7.79 ± 4.04^a^	4.27 ± 3.51^a^	5.79 ± 3.51^b^	5.12 ± 3.51^b^		Detected
BV19	5.64 ± 4.04^a^	4.08 ± 1.58^c^	3.18 ± 2.00^ac^	2.68 ± 1.04^b^	Detected	Detected
BV29	6.32 ± 2.65^b^	4.33 ± 1.51^ab^	5.67 ± 3.57^ab^	5.59 ± 4.11^a^		Detected
BV39	6.80 ± 3.61^a^	4.71 ± 3.51^c^	5.19 ± 4.04^ab^	5.23 ± 3.57^b^		Not detected
BV13	5.16 ± 4.51^ab^	4.17 ± 2.51^b^	4.32 ± 4.00^b^	4.09 ± 3.51^a^		Not detected
BV23	6.74 ± 3.61^a^	4.59 ± 3.11^b^	5.20 ± 3.61^c^	4.87 ± 4.04^ac^		Detected
BV33	7.55 ± 3.06^b^	4.37 ± 3.06^a^	5.12 ± 3.51^a^	5.51 ± 4.51^ab^		Not detected
FDA ML	5.0	2.0	4.0	2.7	0.0	0.0

*Note:* A = Shade A; B = Shade B; V1, 2, or 3 = Vendors 1, 2, or 3; 9 or 3 = sample collected at 9:00 a.m. or 3:00 p.m. Means ± SD with different superscripts in the same row are significantly different at *p* < 0.05.

Abbreviation: Enteric, Enterobacteriaceae.

The presence of high microbial counts in beef samples can be attributed to several factors, including poor hygiene practices during meat processing, inadequate refrigeration, cross‐contamination during handling and transportation, processing methods, and geographical location, coupled with inadequate food safety and hygiene training. High microbial loads at the point of sale significantly elevate foodborne disease risks since some pathogens (*Staphylococcus aureus*) can produce heat‐stable enterotoxins that remain active after cooking [36]. Microbial count also helps to determine good or bad hygiene practices in the slaughterhouses. High enumeration leads to off‐odors, slime, discoloration, and reduced shelf‐life, which may cause reduced consumer confidence and market value. Some of the pathogens (the spore formers) may survive the cooking temperature and later multiply if left in the danger zone for long [37].

The consumption of contaminated beef can lead to outbreaks of foodborne diseases and other health‐related problems and could pose serious health risks to consumers. Sundari et al. [38] stated that microbial counts recorded from beef samples from traditional markets could be higher due to poor hygiene practices and inadequate standard storage facilities and conditions. Shaltout [39] added that the use of different detection methods, such as culture‐based and PCR‐based methods, could have a significant effect on the presence of microflora in beef samples. Several other similar studies reported lower microbial indicator counts than the present study. Kim et al. [40], Mazizi et al. [41], and Sundari et al. [38] recorded 3–4 log CFU/g, 3.3 log CFU/g, and 0–16.7 × 10 CFU/g staphylococci concentration, respectively. While Zulfakar et al. [42] reported higher Enterobacteriaceae (7.05 log CFU/g) counts than ours, Kim et al. [40] reported counts almost similar to this study. Again, the level of *E. coli* counts reported by Jansen et al. [30] was also lower comparatively.

## 4. Conclusion

The study evaluated the presence of foodborne pathogens in beef sold in the Ho Central Market. It was largely revealed that the beef samples were heavily contaminated under all the hygiene indicators (APC, Enterobacteriaceae, staphylococci, and *E. coli*) used. This high rate of contamination recorded might be due to unhygienic facilities used during processing. The molecular characterization reported that almost all the samples were contaminated with *Salmonella* spp. About 25% of the samples showed the presence of *E. coli* O157. The presence of *Salmonella* in the beef is disturbing since it can lead to several severe foodborne illnesses among consumers. Again, the detection in raw beef is of significant public health concern, even though beef is typically consumed well‐cooked. Their presence reflects poor hygienic practices during slaughter and processing and poses risks of cross‐contamination across the value chain. Given the low infectious dose of *E. coli* O157 and the potential for postcooking contamination, the safety of consumers cannot be assured. Cutting boards used for vegetables after raw meat, contaminated knives and surfaces, unwashed hands of food handlers, and flies transferring pathogens to cooked food could lead to cross‐contamination even after beef is thoroughly cooked. Hence, the study recommends that the food safety and hygiene authorities should intensify their supervision and training activities and prosecute beef producers who compromise on food safety and hygiene principles. The authorities should also endeavor to carry out awareness creation as a mandate concerning the dangers associated with compromising food safety protocols. However, further similar research is recommended to employ larger sample sizes to improve statistical power and enhance the generalizability of the findings.

## Author Contributions

F.K.M.: conceptualization, data curation, investigation, and writing—original draft; E.L.: data curation, formal analysis, software, and writing—review and editing; F.O.: conceptualization, data curation, investigation, and writing—original draft; N.K.K.: methodology, supervision, validation, and writing—review and editing; A.A.: conceptualization, resources, supervision, and writing—review and editing; E.B.Q.: methodology, validation, formal analysis, and software; L.E‐M.: validation, formal analysis, software, and writing—review and editing; C.G.: formal analysis, validation, and data curation.

## Funding

No funding was received for this manuscript.

## Conflicts of Interest

The authors declare no conflicts of interest.

## Data Availability

Data sharing is not applicable to this article, as no datasets were generated or analyzed during the current study.
